# Methane Suppresses Microglial Activation Related to Oxidative, Inflammatory, and Apoptotic Injury during Spinal Cord Injury in Rats

**DOI:** 10.1155/2017/2190897

**Published:** 2017-06-27

**Authors:** WeiHeng Wang, Xiaodong Huang, Jian Li, Aijun Sun, Jiangming Yu, Ning Xie, YanHai Xi, Xiaojian Ye

**Affiliations:** ^1^Department of Orthopaedics, Changzheng Hospital Affiliated to the Second Military Medical University, Shanghai 200003, China; ^2^Department of Anesthesiology, Changzheng Hospital Affiliated to the Second Military Medical University, Shanghai 200003, China; ^3^Institute of Biomedical Engineering, Second Military Medical University, Shanghai 200433, China

## Abstract

**Objective:**

We investigated the hypothesis that methane-rich saline (MS) can be used to repair spinal cord injury (SCI) in a rat model through suppressing microglial activation related to oxidative, inflammatory, and apoptotic injury.

**Methods:**

MS was injected intraperitoneally in rats after SCI. Hematoxylin-eosin (HE) staining, oxidative stress, inflammatory parameters, and cell apoptosis were detected 72 h after SCI to determine the optimal dose. Then, we investigated the protective mechanisms and the long-term effects of MS on SCI. HE and microglial activation were observed. Neurological function was evaluated by the Basso, Beattie, and Bresnahan (BBB) scale.

**Results:**

MS can significantly decrease infarct area and inhibit oxidative stress, inflammation, and cell apoptosis 72 h following SCI. The MS protective effect at a dose of 20 ml/kg was better. Moreover, MS can significantly suppress microglial activation related to oxidative and inflammatory injury after SCI and improve hind limb neurological function.

**Conclusion:**

MS could repair SCI and reduce the release of oxidative stress, inflammatory cytokines, and cell apoptosis produced by activated microglia. MS provides a novel and promising strategy for the treatment of SCI.

## 1. Introduction

Spinal cord injury (SCI) has been regarded as a major problem in the medical field for its high disability [1]. According to statistics in the USA, the incidence of SCI reached 54 to 3393 cases/1 million in 2012, and in-hospital mortality remained high compared with that in 1993 [2]. Patients with SCI are often accompanied with severe physical activity disorders, imposing heavy burdens on both families and society. There is currently no effective treatment for SCI. The pathological mechanism of SCI is complex, involving multiple factors such as oxidative stress [3], inflammation [4], and apoptosis [5]. Primary traumatic SCI can induce the release of reactive oxygen species (ROS) and inflammatory factors, triggering the activation of microglia. Microglia are known to be widely located throughout the central nervous system (CNS). They are special glial cells with various different important functions due to the environmental changes in the spinal cord. Excessive activation of microglia can release large amounts of ROS and inflammatory cytokines, which may lead to more serious secondary SCI [6, 7]. Besides releasing large amounts of ROS and inflammatory cytokines, overactive astrocytes can release excessive proteoglycans and intermediate filaments, resulting in glial scar formation [8, 9]. Excessive activation of microglia is regarded as a critical factor increasing secondary SCI and hindering axonal regeneration. Therefore, SCI treatment strategies should not only focus on the removal of injury factors such as spinal surgery to remove mechanical compression but also need to reduce the excessive activation of microglia related to oxidative, inflammatory, and apoptotic injury. Studies have shown that reducing inflammation, oxidative stress, and apoptosis, especially neuronal cell apoptosis, can improve the prognosis of SCI markedly [10–13]. However, the efficacy of currently available drugs, surgery, and other treatments in helping recover the neurological function is limited with various adverse effects, and therefore, better treatment strategies for SCI need to be explored urgently.

Methane is the simplest organism in nature and one of the most important gases that contribute to global warming [14]. Hydrogen produced by human GI tract normal flora can be converted into methane in the intestine [15]. Methane in the human body can freely penetrate cell membranes into organelles. However, the physiological role of methane in the human body has been neglected. In their preliminary study on the physiological role of methane in 2012, Boros et al. [16] reported that the anti-inflammatory and antioxidative effects of methane played an important role in intestinal ischemia/reperfusion injury (IRI). Ye et al. [17] found that methane could suppress inflammation and apoptosis in hepatic IRI. Wu et al. [18] reported that the anti-inflammatory effect of methane on streptozotocin could induce diabetic retinopathy. Wu et al. [18] found that methane could attenuate myocardial ischemia injury due to its antioxidant, antiapoptotic, and anti-inflammatory properties. Although the therapeutic effects of methane on some diseases have been preliminary studied, the methane effect on SCI and related mechanisms have not been reported. In this study, we intended to verify whether intraperitoneal (i.p.) injection with methane-rich saline (MS) could help repair SCI in a rat model through suppressing microglial activation related to oxidative, inflammatory, and apoptotic injury.

## 2. Materials and Methods

### 2.1. Animals

Female Sprague-Dawley (SD) rats (200–220 g) used in this experiment were provided by the Experimental Animal Center of the Second Military Medical University. Rats were given free access to food and water at 20–22°C for 1-2 weeks before surgery to adapt to the environment. All experimental procedures were agreed by the Second Military Medical University Experimental Animal Management Ethics Committee.

### 2.2. MS Production and Detection

Saturated MS was provided by the Department of Navy Aeromedicine of the said university. Using the method described by Ye et al. [17], pure methane (>99.9%) stored in a gas tank (Jixiang Standard Gas Co. Ltd., Shanghai, China) was saturated with 0.4 MPa in saline for 3 h, stored at 4°C, and freshly prepared 24 h prior to administration to the animals to ensure the methane level of injection. According to calculation, the concentration of MS was 0.99 mmol/l. In addition, the degassing of the saturated MS stored at 4°C was inevitable and effective. The concentration of methane in blood of normal and SCI rats was detected by gas chromatography (Gas Chromatography-9860, Qiyang Co. Ltd., Shanghai, China) 10 min after injection. According to the result, there was no significant difference in blood methane concentrations between healthy rats and SCI rats (*P* > 0.05) (in Supplemental Figure 1 available online at https://doi.org/10.1155/2017/2190897).

### 2.3. Establishment of SCI Rat Model

The rats were anesthetized by 10% chloral hydrate 30 ml/kg and then fixed in a bayonet-type rat fixator. The SCI model was induced by dropping a 10 g hammer freely from a 25 cm height on the exposed dura mater at the T9-10 level as described by Tsai et al. [19]. Successful establishment of the SCI modeling was represented by the presence of spinal shock as symbolized by spasmodic back-and-forth swing of the rat tail and hind limbs. To prevent infection, penicillin (500,000 units per day) was administered intramuscularly for 3 consecutive days after surgery. To help urination, the rats received abdominal massage every 12 h after surgery until they were able to void spontaneously. Weight change was observed during the whole experiment. The rats in the sham group received the same operation without being injured by the hammer. Rats without symptoms of spinal shock were excluded.

### 2.4. Experimental Design

This experiment was divided into two parts. In part 1, we test the effect of MS and determine the optimal dose. 50 female SD rats were randomly divided into 5 groups equally (*n* = 10): sham, SCI, SCI + 0.5 ml/kg MS, SCI + 5 ml/kg MS, and SCI + 20 ml/kg MS. Rats in sham and SCI groups were injected i.p. with normal saline (NS), and rats in the three SCI + MS groups received i.p. injection of 0.5, 2, and 20 ml/kg MS, respectively, every 12 h. After 72 h SCI, rats were sacrificed and the injured spinal cord lesions (6 mm around the center) were harvested. The obtained samples were frozen rapidly for further study. In part 2, we studied the protective mechanism of MS on SCI and the long-term effect. Forty-five female SD rats were equally randomized into 3 groups: sham, SCI, and SCI + 20 ml/kg MS. The rats were processed in the same way as described in part 1. After successful establishment of the SCI model, rats were injected *i.p.* with 20 ml/kg MS or NS every 12 h for the next days. The injured spinal cord about 6 mm was harvested 2 weeks after SCI to analyze the hind limb performance by using the Basso, Beattie, and Bresnahan (BBB) scoring system.

### 2.5. Histopathological Observation of SCI

The rats that failed in modeling and died were excluded. 72 hours and 2 weeks after SCI, the rats were anesthetized with overdose chloral hydrate. After perfusion with 0.01 M PBS for 10 min, the rats were perfused with 4% paraformaldehyde (PFA) for 20 min. After protein denaturation, the lamina was opened and the injured segment 3 mm above and below the damaged spinal cord (*n* = 5) was harvested. The removed spinal cord was fixed in 4% PFA for 4 h, alcohol dehydrated, paraffin embedded, cut into 5 *μ*m sections, HE stained, and finally observed under a microscope.

### 2.6. Detection of MAD and SOD

Rats were sacrificed 72 hours after successful modeling, and the spinal cord was harvested and frozen in liquid nitrogen (*n* = 5). The level of malondialdehyde (MDA) in the tissue was taken as the level of lipid oxidation in the tissue, while the superoxide dismutase (SOD) was the index of the tissue antioxidant. The spinal cord tissue (100 *μ*g) was dissolved in 1 ml NS at 4°C, and the supernatant was collected by centrifugation at 2000*g* for 15 min. The SOD activity (U/m) and MAD concentration (nmol/mg) were measured, and the amount of protein in the injured spinal cord was determined by using the BCA kit (Jiancheng Bioengineering Institute, Nanjing, China) according to the manufacturer's instruction.

### 2.7. Detection of Inflammatory Cytokines by Enzyme-Linked Immunosorbent Assay (ELISA)

72 h after SCI, the injured spinal cord was harvested and frozen in liquid nitrogen (*n* = 5). The collected spinal cord tissue was thawed, weighed, and dissolved in 1 ml NS at 4°C, and the homogenate was collected by centrifugation at 2000*g* for 15 min. The spinal cord supernatant was collected, and the content of TNF-*α*, IL-1*β*, and IL-6 in the supernatant were detected strictly according to the instructions of the ELISA test kit (R&D Systems, Minneapolis, MN, USA) by using the kit instructions (ELx800, BioTek) at 450 nm absorbance value.

### 2.8. Western Blot

72 h after SCI, the injured spinal cord was harvested and frozen in liquid nitrogen (*n* = 5). The samples were homogenized in the cell lysate and centrifuged at 14000*g* for 15 mins at 4°C. The supernatant was collected, and the protein concentration was determined by BCA. After adjusting the protein concentration, the sample (50 mg) was denatured in Laemmli buffer for 5 min and loaded onto 12% SDS-PAGE gel. After the transfer of the protein, the nitrocellulose membrane was blocked with TBS containing 5% nonfat dry milk at room temperature for 2 h, washed twice with TBST, incubated overnight in caspase-3 antibody (cell signaling # 9662 1 : 500), washed again 3 times with TBST, and finally incubated with horseradish peroxidase- (HRP-) labelled goat anti-rabbit secondary antibody (Jackson 1 : 2000) at room temperature for 2 h. The membrane was removed and the excess liquid was discarded. The PVDF membrane was wrapped with a plastic wrap and developed in an X-ray film. The ratio of the optical density (OD) of the strip to that of the GAPDH band was calculated as the relative expression level of the sample protein level.

### 2.9. Immunohistochemistry

72 hours and 2 weeks after SCI, the injured spinal cord was harvested and the expression of Iba-1 was detected by immunohistochemistry (*n* = 5). First of all, rats in each group were anesthetized with excess anesthesia and perfused with precooled PBS for 10 min and then with 4% PFA for 20 min. The spinal cord 3 mm above and below the affected segment was removed, fixed in 4% PFA for 4 h, and dehydrated with 20% sucrose for 24 h and then with 30% sucrose for 48 h. After the tissue was settled, the tissue was fixed with OTC tissue fixation, sliced to 10 *μ*m sections, stained immunohistochemically, rinsed with PBS for 5 min × 3, blocked with donkey serum for 2 h, and incubated overnight with primary antibodies goat anti-Iba-1 (1 : 500, Abcam, Cambridge, UK) at room temperature. The tissue was washed again with PBS three times and incubated with the corresponding secondary antibody for 2 h. The nuclei were stained with DAPI. The images were taken with a fluorescence microscope (Olympus, Tokyo, Japan). Image-Pro Plus 6.0 (Media Cybernetics, Silver, Spring, USA) was used to quantify the number of positive cells.

### 2.10. Detection of the Apoptosis Rate by TUNEL Staining

The apoptosis rate of the injured spinal cord was detected by TUNEL staining 72 h after SCI (*n* = 5). The experiment was carried out strictly according to the instructions of TUNEL kit (Roche Molecular Biochemicals, Indianapolis, IN, USA). Image-Pro Plus 6.0 (Media Cybernetics, Silver, Spring, USA) was used to calculate the number of apoptotic cells. 10 slices were randomly selected and the apoptotic index was calculated.

### 2.11. The Hind Limb Motor Function

The rats were placed on the platform before and after 1, 3, 7, 10, and 14 d of SCI. The activity and torso movement of the hip and ankle joints in the three groups (*n* = 15) were observed by using the BBB grading method [20].

### 2.12. Statistical Analysis

Data were analyzed using SPSS 21.0 software (SPSS, Chicago, IL, USA). Measurement data are expressed as mean ± SD. The motor function of the hind limbs was evaluated by repeated measure ANOVA and Fisher's LSD post hoc test. Differences in other indicators between groups were compared by one-way analysis of variance (ANOVA). Values of *P* < 0.05 were considered statistically significant.

## 3. Result

### 3.1. MS Improves the Rat Hind Limb Motor Function Following SCI

**Figure 1:**
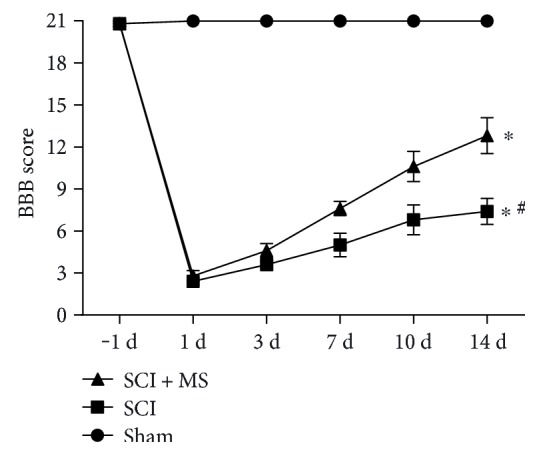
BBB scores were calculated to evaluate the hind limb recovery. All data are expressed as the mean ± sem (*n* = 15). ^∗^*P* < 0.05 versus the sham group. ^#^*P* < 0.05 versus the SCI + MS group.

BBB score was used to evaluate the hind limb recovery in rats after SCI (Figure 1). The BBB scores in the SCI and SCI + MS groups were significantly lower than those in the sham group at 1, 3, 7, 10, and 14 d after surgery (*P* < 0.05), confirming the success in spinal cord injury induction. Animals that received MS had significantly higher BBB scores than those that received physiological saline (*P* < 0.05). In our study, BBB scores increased over time in both SCI and SCI + MS groups, indicating recovery of motor function after SCI in rats. In the SCI + MS group, BBB scores at 14 d time points were significantly higher than those in the SCI group (*P* < 0.05), indicating improved recovery of motor function in rats receiving MS compared with rats receiving NS.

### 3.2. Morphometric Changes

**Figure 2:**
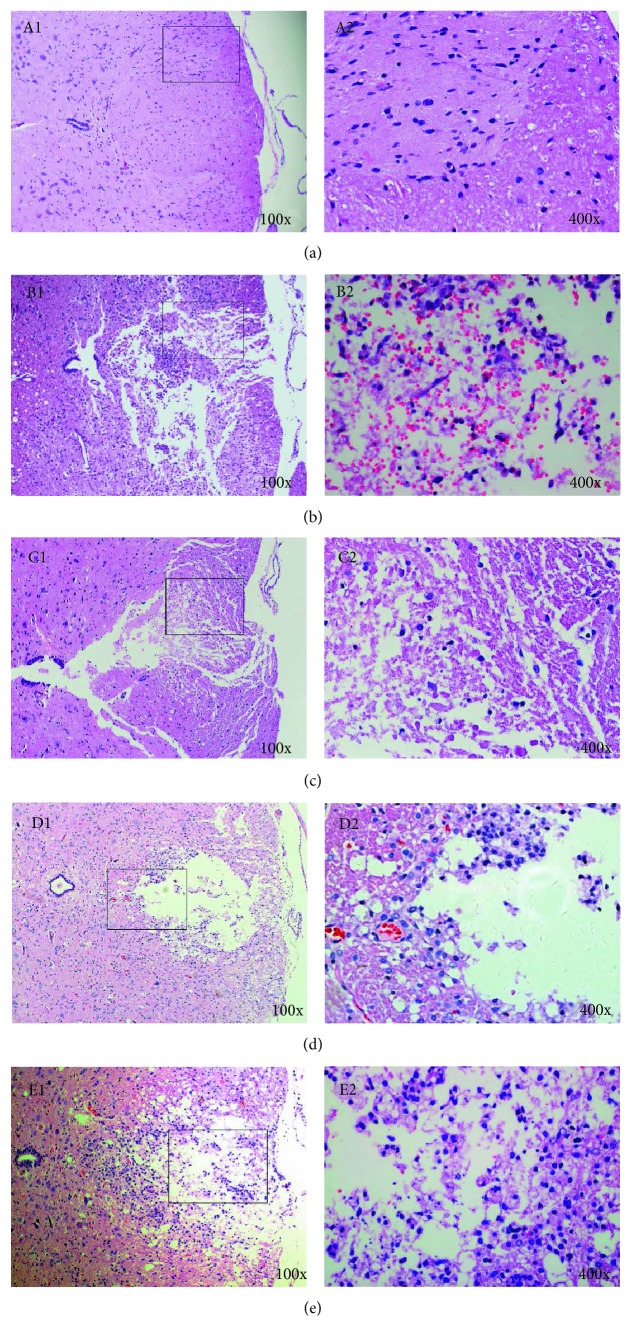
Methane-rich saline treatment attenuated hemorrhage, inflammatory cell infiltration (72 h), and syringomyelia (2 w) at the lesion site. (a) Normal spinal cord; (b) spinal cord at 72 h after SCI; (c) spinal cord in the SCI + 20 ml/kg MS group 72 h after SCI; (d) spinal cord at 14 d after SCI; (e) spinal cord in the SCI + 20 ml/kg MS group at 14 d after SCI.

The normal spinal cord tissue was characterized by a clear boundary between the gray and white matter and a clear outline of neuronal cells with uniform and deep staining of the cytoplasm. The entire spinal cord structure was complete (Figure 2(a)). HE staining 72 h after SCI showed that MS administration significantly reduced cell death, hemorrhage, and inflammatory cell infiltration in the spinal cord. In the SCI group, hemorrhage, liquefaction, and inflammatory cell infiltration were observed in the middle of the spinal cord lesion; the spinal cord boundary between the gray and white matter was not clear; the spinal cord tissue was characterized by disordered nerve fibers accompanied with a large number of necrotic and atrophic neuronal cells; granular and vacuolar degeneration was noticed in the cytoplasm of neuronal cells (Figure 2(b)). In the SCI + 2 ml/kg MS group 72 h after SCI, local hemorrhage and some inflammatory cell infiltration were observed in the injured spinal cord; the structure of neuronal cells was almost complete; some neuronal cells showed vacuolar degeneration and infiltration of some inflammatory cells (Figure 2(c)). In the SCI + 20 ml/kg MS group, there were fewer neuronal and inflammatory cells than those in the SCI+ 2 ml/kg MS group. In the SCI group, spinal cord cavities were observed two weeks after SCI; the spinal cord boundary between the gray and white matter was not clear enough with inflammatory cell infiltration (Figure 2(d)). In the SCI + 20 ml/kg MS group, the cross-sectional area of the spinal cord cavity was small with less inflammatory cell infiltration (Figure 2(e)).

### 3.3. MS Attenuates Oxidative Stress in SCI

**Figure 3:**
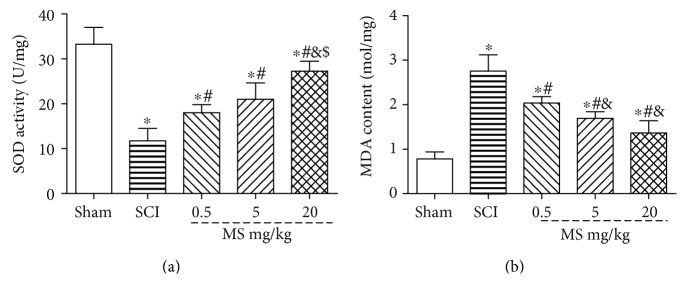
The level of oxidative stress in the spinal cord was assessed by SOD and MDA. (a) MDA content in the spinal cord tissue in the sham, SCI, and SCI + 0.5, 5, and 20 ml/kg MS groups and (b) SOD level in the spinal cord tissue in the sham, SCI, and SCI + 0.5, 5, and 20 ml/kg MS groups. ^∗^*P* < 0.05 versus the sham group; ^#^*P* < 0.05 versus the SCI group; ^&^*P* < 0.05 versus the SCI + 0.5 ml/kg group; ^$^*P* < 0.05 versus the SCI + 5 ml/kg group (*n* = 5).

MDA and SOD levels were measured 72 h after SCI to determine the level of spinal cord oxidative stress (Figure 3). In the SCI group, the SOD level was increased and MDA level was decreased significantly as compared with those in the sham group. In the SCI + 0.5, 5, and 20 ml/kg MS groups, the MDA level was decreased and SOD level was increased significantly as compared with those in the SCI group (both *P* < 0.05). In the SCI + 20 ml/kg MS group, the MDA level was decreased and SOD level was increased significantly as compared with those in the SCI + 0.5 and 5 ml/kg MS groups (both *P* < 0.05). In addition, the protective effect of MS against SCI in the SCI + 20 ml/kg MS groups was better than that in the other two MS groups, indicating that the protective effect of MS against SCI was dose-dependent.

### 3.4. MS Reduces Inflammation in SCI

**Figure 4:**
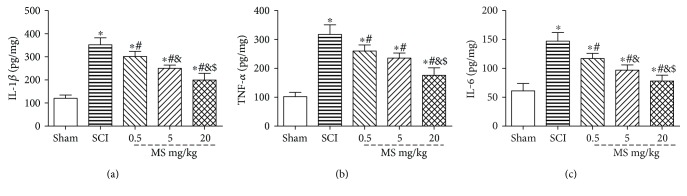
Determination of IL-1*β*, TNF-*α*, and IL-6 levels in the spinal cord tissue by ELISA in part 1. The content of TNF-*α* (a), IL-1*β* (b), and IL-6 (c) in the sham, SCI, SCI + 0.5 ml/kg, SCI + 5 ml/kg, and SCI + 20 ml/kg groups. ^∗^*P* < 0.05 versus the sham group. ^#^*P* < 0.05 versus the SCI group. ^&^*P* < 0.05 versus the SCI + 0.5 ml/kg group. ^$^*P* < 0.05 versus the SCI + 5 ml/kg group (*n* = 5).

The content of IL-1*β*, TNF-*α*, and IL-6 were detected at 72 h after SCI to determine the level of spinal cord inflammatory levels by ELISA (Figure 4()), knowing that TNF-*α*, IL-1*β*, and IL-6 are major indicators of inflammation. The levels of TNF-*α*, IL-1*β*, and IL-6 in SCI rats were increased significantly as compared with those in the sham group (*P* < 0.05). However, the TNF-*α*, IL-1*β*, and IL-6 content was reduced significantly after treatment with MS (*P* < 0.05). In addition, TNF-*α*, IL-1*β*, and IL-6 levels were decreased significantly in the 20 ml/kg MS group as compared with those in the 0.5 and 5 ml/kg MS groups (*P* < 0.05).

### 3.5. MS Suppressed the Apoptosis Following SCI

**Figure 5:**
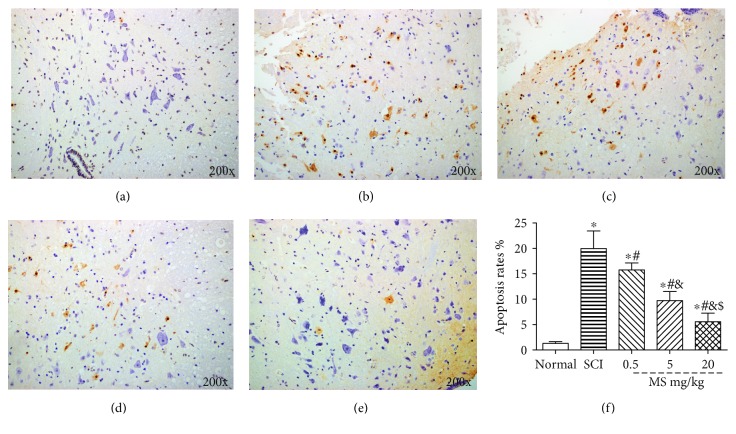
Cell apoptosis in the spinal lesion was investigated by TUNEL staining 72 h following SCI. (a) Spinal cord in the sham group; (b) spinal cord in the SCI group; (c) spinal cord in the SCI+ 0.5 ml/kg MS group; (d) spinal cord in the SCI + 5 ml/kg MS group; (e) spinal cord in the SCI+ 20 ml/kg MS group; (f) apoptosis rate. ^∗^*P* < 0.05 versus the sham group; ^#^*P* < 0.05 versus the SCI group; ^&^*P* < 0.05 versus the SCI + 0.5 ml/kg MS group; ^$^*P* < 0.05 versus the SCI + 5 ml/kg MS group (*n* = 5).

**Figure 6:**
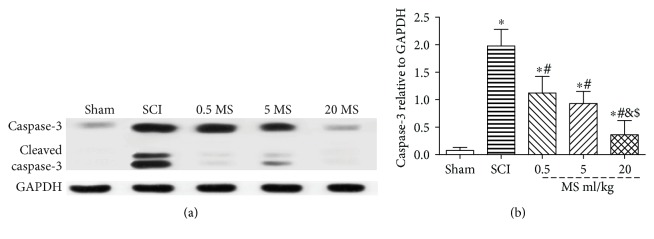
The caspase-3 protein in spinal lesion site was evaluated by Western blot. ^∗^*P* < 0.05 versus the sham group. ^#^*P* < 0.05 versus the SCI group. ^&^*P* < 0.05 versus the SCI + 0.5 ml/kg group. ^$^*P* < 0.05 versus the SCI + 5 ml/kg group (*n* = 5).

MS exerted an antiapoptotic effect on the spinal cord after SCI as demonstrated by TUNEL staining and Western blot. TUNEL stain was used to evaluate the cell apoptosis rate. The caspase-3 protein was invested by Western blot. TUNEL staining showed that the apoptosis rate was increased significantly in the SCI group and decreased markedly in the 20 ml/kg MS group 72 h after SCI (*P* < 0.05) (Figure 5). Western blot assay also showed that the protein content of caspase-3 was increased significantly in the SCI group as compared with that in the sham group. However, caspase-3 protein was reduced significantly in the 20 ml/kg MS group as compared with that in the SCI group (*P* < 0.05) (Figure 6).

The above results demonstrated that MS administration exerted a dose-dependent effect against SCI. Seeing that this protective effect was better in the 20 ml/kg MS group, the dose of 20 ml/kg MS was used in part 2 experiments to investigate its effect in restraining microglial activation and long-term effect against SCI.

### 3.6. MS Suppresses Microglial Activation in SCI

**Figure 7:**
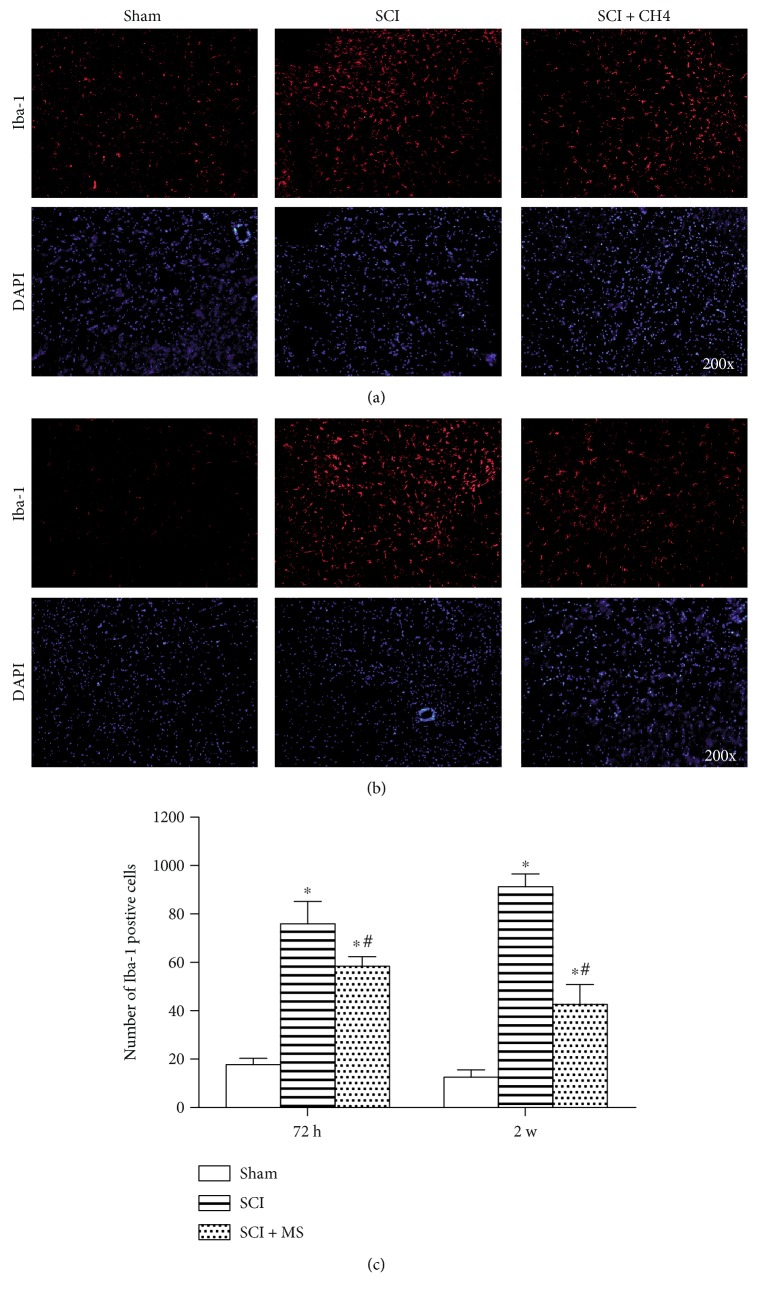
MS administration restrained SCI-induced microglial activation in the spinal cord lesion at (a) 72 h and (b) 2 w after SCI. (c) The number of Iba-1-positive cells at 72 h and 2 w after SCI. MS administration significantly suppressed the microglial activation and alleviated the excessive expression of Iba-1. ^∗^*P* < 0.05 versus the sham group. ^#^*P* < 0.05 versus the SCI group (*n* = 5).

Knowing that the increased number and activation of microglia play a critical role in the development of secondary SCI, we investigated microglial activation by immunohistochemistry at 72 h (Figure 7(a)) and 2 w (Figure 7(b)) after SCI with and without 20 ml/kg MS treatment. It is common knowledge that microglia can specifically express Iba-1. As shown in Figure 7, some Iba-1-positive cells were observed in the sham group, while more Iba-1-positive cells were detected in the spinal cord lesion at 72 h and 2 w after SCI. It was found that the number of Iba-1-positive cells was decreased, and microglial activation was inhibited after administration of 20 ml/kg MS. The morphology was shown by immunofluorescence, and SCI-induced activation of microglia were characteristic of hypertrophy and hyperplasia, and the number of Iba-1-positive cells was dramatically decreased at 72 h and 2 w after SCI (Figure 7(c), *P* < 0.05). The microglial activation and excessive expression of Iba-1 were suppressed significantly after MS administration. In addition, the number of Iba-1-positive cells at 2 w after SCI was decreased more significantly than that at 72 h after SCI (*P* < 0.05).

## 4. Discussion

In recent years, great and rapid progress has been made in the study of biogases such as nitric oxide (NO) [21], carbon monoxide (CO) [22], and hydrogen sulfide (H2S) [23]. Over the last decade, hydrogen has become a hotspot of research due to its antioxidative, anti-inflammatory, and antiapoptotic properties [24–27]. Bacteria in the human gut can produce hydrogen gas, and hydrogen can be converted to methane in the gut by methane-producing bacteria [15]. But the physiological and treatment role of methane in the human body has been overlooked. As we all know, methane is one of the most abundant and easiest alkane organic gases in nature, making contributions to the “greenhouse” effect. Recently, some scholars reported that the methane had positive effects on diabetic retinopathy [18], intestinal IR [28], and liver IR [17] via antioxidative, anti-inflammatory, and antiapoptotic effect [29]. In addition, the diffusible feature of gas molecules including hydrogen, CO, and ethane renders them to penetrate into cell membranes and organelles. What is more, methane can pass through the blood-spinal cord barrier freely. These features of methane provide the possibility of being used for the treatment of SCI.

SCI is characterized by high morbidity and mortality and can be divided into three stages according to neuropathology [30–33]: the first stage is an early stage of inflammatory response, mainly from the beginning of injury to damage after 3 days, and this stage is characterized by bleeding, myelin sheath damage, cell death, tissue edema, and inflammatory cell infiltration. In the first stage, oxidant stress and inflammation are considered to be major factors in SCI. A large number of studies have confirmed that excessive amounts of ROS can lead to oxidative DNA damage, cell damage, and spinal cord dysfunction in the early stages of SCI. Therefore, the content of ROS and MDA can represent the severity of oxidative stress injury in cells [34]. Inflammation is the main reason for the development of SCI. In the SCI first stage of inflammatory response, a large number of inflammatory cells and microglia are activated and release a large number of inflammatory factors, such as TNF-*α*, IL-1*β*, and IL-6, which result in inflammation cascade leading to serious secondary SCI. The second stage is the necrotic material clearance stage, and this stage is from 4 days to 2 weeks after injury, characterized by edema, microglial activation and proliferation, necrotic cell clearance, syringomyelia formation, and apoptosis. Apoptosis is an important factor in cell inactivation. Under physiological conditions, apoptosis is a phenomenon of cell death. In SCI, oxidative stress and inflammatory response can increase the apoptosis rate of the spinal cord [35]. Excessive activation of microglia can produce a variety of inflammatory factors, resulting in imbalance in the immune regulation of the spinal cord and making SCI even worse. The third stage is the stage of glial cell proliferation from 2 weeks after injury, characterized by astroglial proliferation and spinal cord volume reduction [36]. In the third stage of SCI, proliferation and activation of astrocytes can form glial scar, which is the main obstacle to nerve regeneration in the late stage SCI [37, 38].

In our study, we for the first time discovered that MS could repair SCI, attenuate oxidative stress and cell apoptosis, and reduce the release of inflammatory cytokines produced by microglial activation. MS administration i.p. could significantly reduce the content of inflammatory cytokines in the spinal lesion. MS could also significantly increase the SOD activity and decrease the content of MAD, thus reducing the oxidative stress injury to the spinal cord. In terms of apoptosis of the spinal cord tissue, MS inhibited the expression of cleaved caspase-3. In addition, MS administration could modulate immune response and restrain microglial activation in the region affected by SCI-induced SCI. MS could decrease the intensity of inflammatory response and oxidative stress in the injured spinal cord by decreasing microglial overactivation in the injured spinal cord, thus reducing SCI and promoting spinal cord repair. In short, we focused on the pathophysiological processes of inflammation, oxidative stress, and apoptosis during the course of SCI and demonstrated that MS could reduce SCI damage by restraining microglial activation and suppress glial-associated oxidative, inflammatory, and apoptotic damage.

In the first part, we studied the effects of different doses of methane on the early stage of SCI. We examined changes in oxidative stress in the spinal cord by examining the content of SOD and MDA in the injured spinal cord tissue 24 h after SCI and examined the levels of TNF-*α*, IL-1*β*, and IL-6 by ELISA to determine the intensity of inflammatory response in SCI. Our results confirmed that oxidant and inflammation levels were increased dramatically 72 h after SCI and that MS could downregulate oxidant stress and the content of inflammatory factors 72 h after SCI. The results showed that different concentrations of MS could all attenuate the inflammatory and oxidative stress induced by SCI, which may be due to decreased inflammatory cytokines and ROS. Various types of cell death are known to be related to the development of SCI. Studies have shown that reducing apoptosis, especially neuronal cell apoptosis, could significantly improve the prognosis of SCI [39]. In the present study, apoptosis was measured in 72 h in rats treated with or without MS injection. It was found that i.p. injection of 20 ml/kg MS significantly inhibited apoptosis. This may be the mechanism by which MS exerts its protective effect. This effect was dose-dependent and was most pronounced at a dose of 20 ml/kg. Therefore, 20 ml/kg MS was utilized in part 2 experiments to estimate the protective mechanism and long-term effect. Microglial activation was detected at 72 h and 2 w after SCI. It was found that microglia in the SCI region were activated at 72 h and 2 w, and this microglial activation could be attenuated by MS administration. Inhibition of microglial overactivation could reduce the intensity of inflammatory response, oxidative stress, and apoptosis in the spinal cord, suggesting that methane exerted its protective effect against SCI by suppressing the microglial activation related to oxidative, inflammatory, and apoptosis injury after SCI.

In terms of the motor function after SCI in the rats, we further investigated BBB scores for 2 weeks after SCI in rats with and without MS, knowing that it has more clinical significance. The results showed that treatment with MS after SCI helped improve the motor function, especially in the early stage of SCI, confirming that the earlier the MS is administered the better the protective effect would be. However, due to the limited number of animals (*n* = 15) and the subjective nature of the BBB scoring system used in this study, there may be some errors in the experimental results.

However, the exact molecular mechanism underlying the suppressive effect of methane on microglial activation related to oxidative stress, apoptosis, and inflammation response remains unclear. Different researchers have proposed different hypotheses. Boros et al. [16] assumed that methane may accumulate at the interfaces of cell membranes and alter the physicochemical properties or the situ functionality of proteins embedded in the environment. Kai et al. [40] proposed that methane may affect membrane pathways, affect G protein, membrane or receptor-mediated signaling and acetylcholine-activated ion channel kinetics. Fink [41] proposed that more studies should be performed to elucidate the existence of the oxygenase which can use methane as a substrate, the methane receptor in cell, and the relationship between methanol, reactive alcohol, NAD(P)+/NAD(P)H ratio, and methane. More studies are required to confirm the effect of MS on microglial activation before it can be used in clinical practice [42]. We hypothesize that methane might have the effect of regulating the classical (M1) and alternative (M2) balance of microglia and promoting microglia M1 conversion to M2 in SCI. Further research is needed to elucidate the association between methane and microglia cell typing and the molecular mechanism.

## 5. Conclusion

The present study demonstrated that methane could significantly suppress microglial activation related to oxidative, inflammatory, and apoptotic injury following SCI. Taken together, methane is promising for the clinical treatment of SCI for its nontoxicity, relative stability, and free permeability through cell membranes. The effect of manner and the route of methane administration on SCI and the exact molecular mechanism of methane need to be further investigated.

## Supplementary Material

Supplementary figure 1: the concentration of methane in blood of normal and SCI rats 10 min after injection.

## References

[1] Selvarajah S., Hammond E. R., Schneider E. B. (2015). Trends in traumatic spinal cord injury. *Jama*.

[2] Jain N. B., Ayers G. D., Peterson E. N. (2015). Traumatic spinal cord injury in the United States, 1993-2012. *Jama*.

[3] Su M., Guan H., Zhang F., Gao Y., Teng X., Yang W. (2016). HDAC6 regulates the chaperone-mediated autophagy to prevent oxidative damage in injured neurons after experimental spinal cord injury. *Oxidative Medicine and Cellular Longevity*.

[4] Fleming J. C., Norenberg M. D., Ramsay D. A. (2006). The cellular inflammatory response in human spinal cords after injury. *Brain: A Journal of Neurology*.

[5] Li C. M., Xie S. J., Wang T., Du W. B., Yang Z. B., Quan R. F. (2015). Effects of electro-acupuncture on neuronal apoptosis and associative function in rats with spinal cord injury. *Zhongguo Gu Shang = China Journal of Orthopaedics and Traumatology*.

[6] Won K. A., Kim M. J., Yang K. Y. (2014). The glial-neuronal GRK2 pathway participates in the development of trigeminal neuropathic pain in rats. *The Journal of Pain: Official Journal of the American Pain Society*.

[7] Zhao W., Beers D. R., Henkel J. S. (2010). Extracellular mutant SOD1 induces microglial-mediated motoneuron injury. *Glia*.

[8] Pekny M., Pekna M. (2004). Astrocyte intermediate filaments in CNS pathologies and regeneration. *The Journal of Pathology*.

[9] Cregg J. M., Depaul M. A., Filous A. R., Lang B. T., Tran A., Silver J. (2014). Functional regeneration beyond the glial scar. *Experimental Neurology*.

[10] De Rivero Vaccari J. P., Dietrich W. D., Keane R. W. (2016). Therapeutics targeting the inflammasome after central nervous system injury. *Translational Research : The Journal of Laboratory and Clinical Medicine*.

[11] Estrada V., Muller H. W. (2014). Spinal cord injury - there is not just one way of treating it. *F1000prime Reports*.

[12] Mallory G. W., Grahn P. J., Hachmann J. T., Lujan J. L., Lee K. H. (2015). Optical stimulation for restoration of motor function after spinal cord injury. *Mayo Clinic Proceedings*.

[13] Wang J., Pearse D. D. (2015). Therapeutic hypothermia in spinal cord injury: the status of its use and open questions. *International Journal of Molecular Sciences*.

[14] Wang C., Huang G., Liang Z. (2002). Advances in the research on sources and sinks of CH4 and CH4 oxidation (uptake) in soil. *Ying Yong Sheng tai Xue Bao = the Journal of Applied Ecology / Zhongguo Sheng tai Xue Xue hui, Zhongguo Ke Xue Yuan Shenyang Ying Yong Sheng tai Yan Jiu Suo Zhu ban*.

[15] Cloarec D., Bornet F., Gouilloud S., Barry J. L., Salim B., Galmiche J. P. (1990). Breath hydrogen response to lactulose in healthy subjects: relationship to methane producing status. *Gut*.

[16] Boros M., Ghyczy M., Érces D. (2012). The anti-inflammatory effects of methane. *Critical Care Medicine*.

[17] Ye Z., Chen O., Zhang R. (2015). Methane attenuates hepatic ischemia/reperfusion injury in rats through antiapoptotic, anti-inflammatory, and antioxidative actions. *Shock (Augusta, Ga)*.

[18] Wu J., Wang R., Ye Z. (2015). Protective effects of methane-rich saline on diabetic retinopathy via anti-inflammation in a streptozotocin-induced diabetic rat model. *Biochemical and Biophysical Research Communications*.

[19] Tsai M. J., Liao J. F., Lin D. Y. (2010). Silymarin protects spinal cord and cortical cells against oxidative stress and lipopolysaccharide stimulation. *Neurochemistry International*.

[20] Basso D. M., Beattie M. S., Bresnahan J. C. (1996). Graded histological and locomotor outcomes after spinal cord contusion using the NYU weight-drop device versus transection. *Experimental Neurology*.

[21] Stamler J. S. (1994). Redox signaling: nitrosylation and related target interactions of nitric oxide. *Cell*.

[22] Kajimura M., Fukuda R., Bateman R. M., Yamamoto T., Suematsu M. (2010). Interactions of multiple gas-transducing systems: hallmarks and uncertainties of CO, NO, and H_2_S gas biology. *Antioxidants & Redox Signaling*.

[23] Yang X., Hao D., Zhang H., Liu B., Yang M., He B. (2017). Treatment with hydrogen sulfide attenuates sublesional skeletal deterioration following motor complete spinal cord injury in rats. *Osteoporosis International*.

[24] Xie K., Yu Y., Pei Y. (2010). Protective effects of hydrogen gas on murine polymicrobial sepsis via reducing oxidative stress and HMGB1 release. *Shock (Augusta, Ga)*.

[25] Chen X., Liu Q., Wang D. (2015). Protective effects of hydrogen-rich saline on rats with smoke inhalation injury. *Oxidative Medicine and Cellular Longevity*.

[26] Zhao L. L., Hu G. C., Zhu S. S., Li J. F., Liu G. J. (2014). Propofol pretreatment attenuates lipopolysaccharide-induced acute lung injury in rats by activating the phosphoinositide-3-kinase/Akt pathway. *Brazilian Journal of Medical and Biological Research*.

[27] Ohsawa I., Ishikawa M., Takahashi K. (2007). Hydrogen acts as a therapeutic antioxidant by selectively reducing cytotoxic oxygen radicals. *Nature Medicine*.

[28] Chen O., Ye Z., Cao Z. (2016). Methane attenuates myocardial ischemia injury in rats through anti-oxidative, anti-apoptotic and anti-inflammatory actions. *Free Radical Biology and Medicine*.

[29] Song K., Zhang M., Hu J. (2015). Methane-rich saline attenuates ischemia/reperfusion injury of abdominal skin flaps in rats via regulating apoptosis level. *BMC Surgery*.

[30] Kim D. H., Heo S. D., Ahn M. J., Sim K. B., Shin T. K. (2003). Activation of embryonic intermediate filaments contributes to glial scar formation after spinal cord injury in rats. *Journal of Veterinary Science*.

[31] Song M. S., Seo H. S., Yang M. (2009). Activation of Ca^2+^/calmodulin-dependent protein kinase II α in the spinal cords of rats with clip compression injury. *Brain Research*.

[32] Ahn M., Lee C., Jung K. (2012). Immunohistochemical study of arginase-1 in the spinal cords of rats with clip compression injury. *Brain Research*.

[33] Shin T. (2007). Increases in the phosphorylated form of caveolin-1 in the spinal cord of rats with clip compression injury. *Brain Research*.

[34] Tabak O., Gelisgen R., Erman H. (2011). Oxidative lipid, protein, and DNA damage as oxidative stress markers in vascular complications of diabetes mellitus. *Clinical and Investigative Medicine Medecine Clinique et Experimentale*.

[35] Samantaray S., Das A., Matzelle D. C. (2016). Administration of low dose-estrogen attenuates gliosis and protects neurons in acute spinal cord injury in rats. *Journal of Neurochemistry*.

[36] Jung K., Sim K. B., Ahn M., Kim H., Cheong J., Shin T. (2003). Upregulation of phospholipase D1 in the spinal cords of rats with clip compression injury. *Neuroscience Letters*.

[37] Buffo A., Rolando C., Ceruti S. (2010). Astrocytes in the damaged brain: molecular and cellular insights into their reactive response and healing potential. *Biochemical Pharmacology*.

[38] Colangelo A. M., Cirillo G., Lavitrano M. L., Alberghina L., Papa M. (2012). Targeting reactive astrogliosis by novel biotechnological strategies. *Biotechnology Advances*.

[39] de Lavor M. S., Binda N. S., Fukushima F. B. (2015). Ischemia-reperfusion model in rat spinal cord: cell viability and apoptosis signaling study. *International Journal of Clinical and Experimental Pathology*.

[40] Kai T., Jones K. A., Warner D. O. (1998). Halothane attenuates calcium sensitization in airway smooth muscle by inhibiting G-proteins. *Anesthesiology*.

[41] Fink M. P. (2012). Pharmacological effects of inhaled methane: plausible or not?. *Critical Care Medicine*.

[42] Liu W., Wang D., Tao H., Sun X. (2012). Is methane a new therapeutic gas?. *Medical Gas Research*.

