# Supplementary material for: Methane Suppresses Microglial Activation Related to Oxidative, Inflammatory, and Apoptotic Injury during Spinal Cord Injury in Rats
Source: Oxid Med Cell Longev. 2017 Jun 27;2017:2190897. doi: 10.1155/2017/2190897 (PMC5504966; doi:10.1155/2017/2190897)
Supplement: Supplementary file 1 — Supplementary figure 1: the concentration of methane in blood of normal and SCI rats 10 min after injection. [file 2190897.f1.docx]

Supplementary figure.1: the concentration of methane in blood of normal and SCI rats 10 min after injection.
